# Differential sexual network connectivity offers a parsimonious explanation for population-level variations in the prevalence of bacterial vaginosis: a data-driven, model-supported hypothesis

**DOI:** 10.1186/s12905-018-0703-0

**Published:** 2019-01-10

**Authors:** Chris R. Kenyon, Wim Delva, Rebecca M. Brotman

**Affiliations:** 1 STI Unit, Institute of Tropical Medicine, Nationalestraat 155, 2000 Antwerp, Belgium; 2 The South African DST-NRF Centre of Excellence in Epidemiological, Modelling and Analysis (SACEMA), Stellenbosch, South Africa; 3 Department of Global Health, Faculty of Medicine and Health Sciences, Stellenbosch University, Stellenbosch, South Africa; 4 International Centre for Reproductive Health, Ghent University, Ghent, Belgium; 5 Center for Statistics, Hasselt University, Diepenbeek, Belgium; 6 Rega Institute for Medical Research, KU Leuven, Leuven, Belgium; 7 Department of Epidemiology and Public Health, Institute for Genome Sciences, University of Maryland School of Medicine, Ghent, Belgium

**Keywords:** Bacterial vaginosis, Microbiome, Sexual network connectivity, Concurrency, STI, HIV

## Abstract

**Background:**

The prevalence of bacterial vaginosis (BV) and vaginal microbiota types varies dramatically between different populations around the world. Understanding what underpins these differences is important, as high-diversity microbiotas associated with BV are implicated in adverse pregnancy outcomes and enhanced susceptibility to and transmission of sexually transmitted infections.

**Main text:**

We hypothesize that these variations in the vaginal microbiota can, in part, be explained by variations in the connectivity of sexual networks. We argue: 1) Couple-level data suggest that BV-associated bacteria can be sexually transmitted and hence high sexual network connectivity would be expected to promote the spread of BV-associated bacteria. Epidemiological studies have found positive associations between indicators of network connectivity and the prevalence of BV; 2) The relationship between BV prevalence and STI incidence/prevalence can be parsimoniously explained by differential network connectivity; 3) Studies from other mammals are generally supportive of the association between network connectivity and high-diversity vaginal microbiota.

**Conclusion:**

To test this hypothesis, we propose a combination of empirical and simulation-based study designs.

**Electronic supplementary material:**

The online version of this article (10.1186/s12905-018-0703-0) contains supplementary material, which is available to authorized users.

## Background

**Fig. 1:**
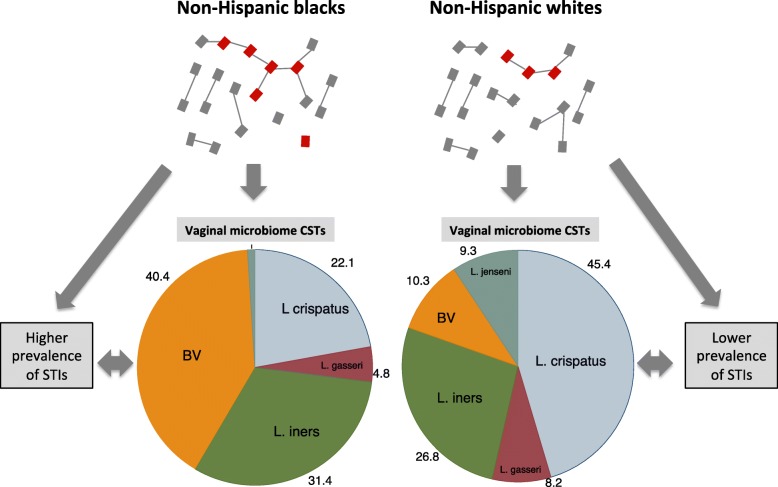
Schematic illustration of interactions between sexual network connectivity, frequency distribution of vaginal community state types (CSTs) and the prevalence of STIs using the example of non-Hispanic blacks and non-Hispanic whites in the USA. Non-Hispanic blacks have been noted to have higher network connectivity - largely due to a high prevalence of sexual partner concurrency [67]. This enhanced network connectivity facilitates the spread of STIs as well as the bacteria responsible for bacterial vaginosis (BV) and possibly less resilient *L. iners* vaginal community state types. BV and the STIs then further facilitate the spread of one another. (The distribution of vaginal CSTs is taken from a study by Ravel et al. [4], the prevalence ratios of STIs are taken from [67, 77]. The community state types are described by the presence of BV or the predominant Lactobacillus species present e.g. ‘*L. iners’* refers to a *Lactobacillus iners* dominant type. The numbers around the pie-charts denote the percent each CST comprises)

Over 17 studies from around the world have established that women’s vaginal microbiota (VMB) can be classified into one of 3 to 9 clusters or community state types (CSTs) [1–3]. The most commonly referenced typing system is that developed by Ravel et al. in 2011 [4]. This schema describes 5 CSTs of which four CSTs were respectively dominated by 4 different *Lactobacillus* species – *L. crispatus, L gasseri, L. iners* and *L. jensenii* (Fig. 1). The fifth CST was characterized by a paucity of *Lactobacillus* spp. and an abundance of a highly diverse polymicrobial community of facultative anaerobic BV-associated bacteria (BVAB), including *Gardnerella vaginalis, Atopobium vaginae, Clostridiales* spp., *Megasphaera* spp. and *Leptotrichia/Sneathia* spp. [1, 5]. This CST (which can be split into two different CSTs [6]) corresponds closely with bacterial vaginosis (BV) as defined by Nugent’s scoring system, as well as pH, and we refer to it here as the BV-VMB. Several longitudinal VMB studies have concluded that the VMB can be relatively stable over time [1, 7, 8]. In a minority of women and particularly those with BV-VMB, the community composition of the VMB can be fairly dynamic [1, 7].

There is little consensus as to why the proportion of reproductive-age women with particular CSTs, and in particular those with BV-VMB, varies so dramatically between different populations [1]. Some have postulated that genetic differences between populations (such as differences in innate and adaptive immune systems, the composition and quantity of vaginal secretions, and ligands on epithelial cell surfaces [4, 9]) may be responsible. Others have argued that the prevalence of practices known to influence the VMB may be responsible: including use of vaginal douching, nutrition, smoking, personal hygiene, methods of birth control, and sexual behaviors [10–12].

Both BV and some *L. iners*-dominated VMBs have been associated with various adverse outcomes, including enhanced susceptibility to STIs such as HIV, enhanced transmission of HIV, pelvic inflammatory disease and a range of adverse pregnancy outcomes [13–15]. One meta-analysis found that BV-VMB increased the risk of HIV acquisition by 60% (relative risk 95% CI 1.2–2.1) [16]. In populations where the prevalence of BV is over 30%, this may translate into 15% of HIV infections being attributable to BV-VMB [16]. Similar lines of reasoning suggest that BV-VMB may explain a considerable proportion of the differential spread of other STIs between different populations [17–23].

We hypothesize that variations in sexual network connectivity may be at least partially responsible for variations in the prevalence of BV-VMB between different populations, and below we present three types of evidence in support of this thesis:

## Individual and couple-level data

Individual and couple-level data suggest that BVAB can be sexually transmitted. Multiple studies have found an association between BV and a number of individual-level sexual risk factors amongst women including: i) a higher number of lifetime sexual partners [24]; ii) retaining the same sexual partner after a diagnosis of BV [25, 26]; iii) a high frequency of vaginal intercourse [27]; iv) self-identification as sex workers [28, 29]; v) anal or receptive oral sex [30, 31]; vi) sharing sex toys between women who have sex with women [32]. Male circumcision has been shown in a randomized controlled trial to result in a 50% lower risk of BV for partners of circumcised males [33]. Circumcision was also associated with a reduction in a wide range of anaerobes in the coronal sulcus including a number of BVAB [33, 34]. Consistent condom use has been shown in a meta-analysis to be associated with a reduced odds for BV [24] and several, but not all studies, have found that inconsistent or no condom use is a risk factor for recurrent BV [24, 27, 35]. These findings suggest that BV can be sexually transmitted. Other non-sexual epidemiologic risk factors for BV include: i) lack of hormonal contraception ii) cigarette smoking, iii) douching [13] (Table 1).

**Table 1:** Correlation of bacterial species between vagina and penile skin and male urethra from couples with bacterial vaginosis and couples without bacterial vaginosis in cross sectional study by Zozaya et al. [39] Only the top 13 most correlated species are shown

	Vagina-Penile Skin	Vagina-Male Urethra
	Rho ^a^	Rho ^a^
Couples with BV (*n* = 65)		
Megasphaera2	**0.549**	0.085
Pv.123-f2–42	**0.537**	**0.584**
Pv.123-f-110	**0.482**	**0.448**
BVAB1	**0.477**	0.153
P.bivia	**0.422**	**0.510**
Prevotella	**0.421**	**0.402**
Gardnerella	**0.419**	**0.324**
Aerococcus	**0.413**	**0.421**
Pv.123-b-95	**0.411**	0.239
L.iners	**0.399**	0.215
Porphyromonas	**0.399**	0.105
Sneathia	**0.376**	**0.258**
Leptotrichia	**0.371**	**0.376**
Couples without BV (*n* = 31)		
Pv.123-f-82	**0.504**	−0.033
Dialister	**0.443**	0.240
L.crispatus	**0.391**	−0.084
L.jensenii	**0.384**	−0.259
Lactobacillus sp.	0.327	0.284
Pv.123-b-46	0.280	**0.379**
Streptococcus	0.221	0.179
U.urealyticum	0.156	−0.071
L.helveticus	0.142	0.223
L.gasseri	0.125	−0.133
Peptoniphilus	0.049	−0.023
Gardnerella	0.034	−0.146
L.iners	−0.020	0.043

^a^Rhos in bold indicate a *P*-value of < 0.05

Couple studies have found high rates of concordance (up to 100%) for various BVAB (including biofilm forming *G. vaginalis* [36]) in women’s VMB and the coronal sulci/distal urethras of their male partners [36–41] or the vaginas of their female partners [42]. A study of the genital microbiomes of 165 men and their partners in Rakai, Uganda found that the penile microbiomes could be segregated into two main groups - a BV and a non-BV-type group [43]. The BV-type group had a higher prevalence and abundance of BV associated bacteria. The female partners of this group were also more likely to have BV [43] as assessed by Nugent’s scoring (the women in this study did not have their vaginal microbiomes characterized molecularly). Two studies that simultaneously characterized the genital microbiomes of women and their male partners found a strong intra-couple correlation for the presence or absence of individual bacterial species [39, 44]. In both studies, concentrations of BVAB were low or undetectable in women without BV and their partners but abundant and concordant at a species level for women with BV and their partners coronal sulci (and to a lesser extent urethra) [39, 44]. Other studies have established a high degree of concordance of oligotype and phenotype (such a biofilm forming or not) of *G. vaginalis* and other BVAB between monogamous partners [36, 45, 46]. In one of these studies, for example, all women with BV had evidence of a biofilm- forming *G. vaginalis* vaginal infection as did all their male partners in their urine [36]. Women who have sex with women have also been shown to have a high degree of concordance for BV status and this has been linked to practices that transmit vaginal fluid between women [47, 48].

STIs are transmitted along sexual networks, and as a result, the amount of connectivity between individuals in the network determines the speed and extent of STI spread in the network [49–51]. Network connectivity is a complex concept that can be characterized by a multi-dimensional array of metrics, including the number of partners per unit time, prevalence of concurrent partnering, size of core groups, type of sex, size of sexual network, length of gaps between partnerships, degree and type of homophily and relations between core and non-core groups [50, 52–57]. We will confine our further consideration of network connectivity to number of partners per unit time and partner concurrency (partnerships overlapping in time). Both of these variables have clear definitions, have multiple prevalence estimates from around the world and have been found to be risk factors for most major STIs, including BV at the individual level [24, 52, 58–61]. We acknowledge however that these variables are measures of local sexual networks. Future work could benefit from incorporating better measures of global sexual network connectivity such as the size of the forward reachable set. Since sexual network connectivity is a population-level property, ecological studies are also necessary to explore the possible explanations for variations in STI prevalence [50, 62]. Although not all studies have reached this conclusion, [63, 64] studies have generally found a positive association between STI prevalence and the prevalence of partner concurrency and/or numbers of partners per unit time [50, 52, 58, 65–67].

**Fig. 2:**
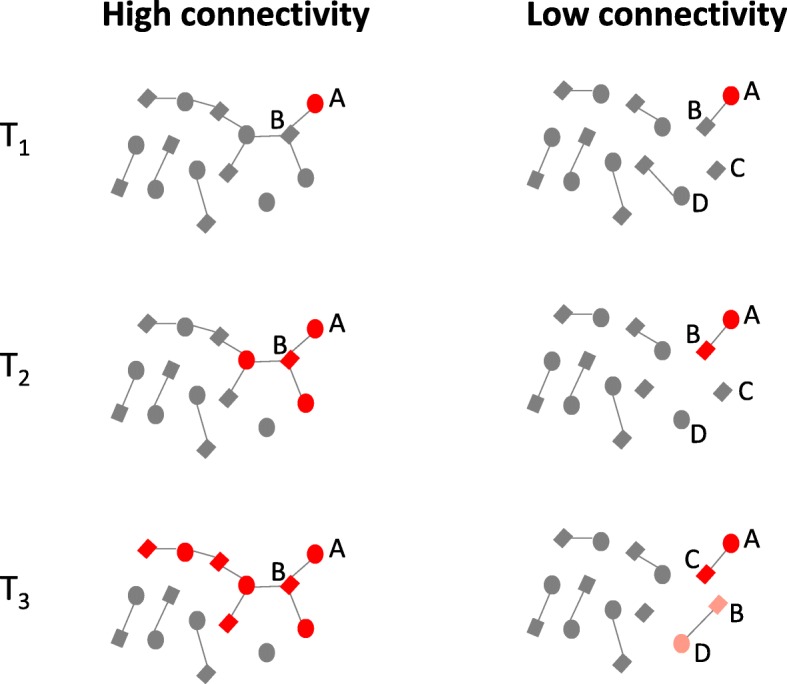
Schematic illustration of how high sexual network connectivity can enhance transmission of bacterial vaginosis associated bacteria (BVAB - depicted in red). In the *low connectivity network* (right), the BVAB are trapped in the A-B relationship until this breaks up when woman A can transmit the BVAB to her new partner (C). Man B may then also transmit BVAB to his new partner (D) but if the gap between his old and new partner exceeds the duration of penile colonization for BVAB (time between T_2_ and T_3_) then he will not transmit to his new partner. In the *high connectivity network* (left), the BVAB does not need to wait for the A-B partnership to end and can, without impediment, spread to other individuals connected via sexual partnerships (Squares-men, Circles-women, Red/Gray nodes-BVAB−/non-BVAB -containing genital microbiome, Gray lines-sexual partnership active on first day of the month; see text for further details)

If BVAB are sexually transmitted then the various ways whereby enhanced network connectivity has been shown to enhance the transmission of various STIs could also promote the spread of BVAB [58]. This is illustrated in Fig. 2 and Additional file 1, which contrast the transmissibility of BVAB in high and low connectivity populations. In Fig. 2, both networks commence with woman ‘A’ having a BV-VMB (red). In the high connectivity network, the BVAB can be transmitted to her partner ‘B’ who can then transmit them to the other women he is having sex with and the BV-VMB can thereby spread through the population. In the low connectivity network, the BVAB are trapped in the ‘A-B’ relationship until it breaks up at T_3_ when both ‘A’ and ‘B’ can transmit the BVAB to their new partners.

Enhanced network connectivity may play a particularly important role in facilitating the spread of the BV-associated *G. vaginalis* and *T. vaginalis*, because of their relatively short periods of colonization in men [68]. *T. vaginalis* colonizes men for only 6 weeks (and women for 60 weeks) [69, 70]. This gender gap in colonization-duration means that *T. vaginalis* would go extinct in a serial monogamous population with at least 7 weeks between consecutive partners of men [66]. Concurrency enables *T. vaginalis* to bypass this bottleneck and thereby could facilitate the spread of *T. vaginalis* and indirectly BV (because *T. vaginalis* predisposes to BV [71]). Similarly, *G. vaginalis* – one of the likely driver species of BV [72, 73] – has been shown to be well adapted to long-term colonization of the high glycogen vaginal environment [74] but poorly suited to long-term urethral colonization in men [74]. *G. vaginalis* thus colonizes men for under 3 months but persists long term in women [36, 75]. One study, for example, found an identical strain of biofilm forming *G. vaginalis* persisting in one woman’s vagina for 15 years [36]. If the BVAB (or at least a number of the keystone species responsible for BV) have a duration of colonization in the male that is as short as that of *G. vaginalis,* then this would result in a break of transmission of BVAB in populations with serial monogamy and gaps of a few months between sexual relationships. The illustration in Fig. 2 would then need to be adapted as ‘B’ would no longer be able to transmit the BVAB to his new partner at T_3_ if the time gap since his previous relationship was long enough to have removed the BVAB from his penile microbiome.

### An individual-based network model of BV transmission

To demonstrate the interacting effects of higher sexual network connectivity and a shorter duration of colonization in men more explicitly, we developed a simple, didactic individual-based model representing two adjacent but entirely separate communities (see Additional file 1: NetworkModelDescription for a detailed description of the model). In the first community only serially monogamous relationships can be formed. In the second, both men and women remain available to form new relationships, regardless of the number of relationships they are already engaged in. In both communities, the duration of BVAB colonization is set to a fixed period of 6 weeks in men and 60 weeks in women. Additional file 2: NetworkMovie shows model output over 10 years with populations of 250 people in each community. In the community where concurrent relationships were allowed, the prevalence of partner concurrency varied between 2 and 11% over the 10-year simulation period, and by the end of it, 42% more relationships had been formed, compared to the community with lower network connectivity. The prevalence of BV-VMB plateaued around 55% in the high connectivity network and around 15% in the low connectivity network (a relative difference of 267%). This is an example based in heterosexual couplings and certainly future models should include partnerships between women as well.


**Additional file 2:** A movie file illustrating the main findings of the Netlogo model of BV transmission. (MP4 60657 kb)


### Epidemiological studies confirm that concurrency and partner number are risk factors for BV

Epidemiological studies have shown number of partners per unit time to be a risk factor for BV-VMB [24, 76, 77]. Partner concurrency has also been shown to be an independent risk factor for BV in a longitudinal study of 3620 women followed up quarterly for 5 visits [58]. In the Rakai study mentioned above, men with extramarital partners were also found to be more likely to have the BV-type penile microbiome than men with no extramarital partners [43]. Finally, an ecological study found associations at the level of countries between the prevalence of male concurrency and the prevalence of BV among women [78]. The same association was found at the level of ethnic groups within countries [78].

## Network connectivity is a parsimonious explanation for the association between the prevalence of BV and STIs

It has been argued that certain racial groups such as ‘black populations’ [9] tend to have a higher prevalence of BV and that this is due to biological differences in susceptibility to BV [4, 9, 79]. The available evidence, however, suggests that populations (irrespective of race) with high network connectivity have a higher BV prevalence. (i) Black populations with low risk behavior as established by low prevalence of HIV and other STIs have low prevalences of BV [80, 81]. (ii) Populations with high network connectivity (as deduced by high STI prevalences) have high BV prevalences regardless of ethnicity. This has been most clearly established in sex workers where sex workers from all ethnic groups with available data have high BV prevalences [2, 28, 29]. BV prevalences have also been found to vary between non-black ethnic/racial groups within Canada, Peru, China and Spain [80]. In the case of Canada and Peru, the high BV-prevalence ethnic groups were also found to have higher prevalences of other STIs suggesting a common risk factor may be responsible [80]. (iii) The available evidence suggests that prior to sexual debut, there are no differences in VMB between ethnic groups but that differences only emerge post debut [82, 83]. (iv) In a longitudinal study, white women with black partners had the same BV prevalence as black women [84] and black women with white partners had the same BV prevalence as white women (C Kenyon’s unpublished data). (v) The VMBs from all racial/ethnic groups profiled thus far include all the major CSTs. It is merely the proportionate mix of CSTs that varies between ethnic groups [1, 85]. (vi) We have been unable to find any published studies that have established evidence of genetic differences in susceptibility to particular VMBs by race/ethnic group.

Populations with a higher prevalence of BV also tend to have a higher prevalence of other STIs [65]. This association has been shown between BV and HIV prevalence at the level of world regions [80], countries [65] and ethnic groups within countries (Fig. 1) [80]. These positive associations between STIs and BV could be due to a common underlying risk factor (such as network connectivity [65]) Alternatively, they could be explained by these STIs enhancing the probability of transition to BV [17]. However, this explanation begs the question, why did these populations have higher STI prevalences? A possible answer is that they have poorer STI treatment services [86]. The correlation between STI prevalence and quality of STI services is, however, weak or absent [87]. Furthermore, this does not explain the strong correlation between the incurable STI, herpes simplex virus-2 (HSV-2), and BV and other STIs [88, 89]. These considerations lead us to predict that the most parsimonious explanation for why the prevalence of BV and various STIs is higher in certain populations than others is that these populations have more densely connected sexual networks. High network connectivity would be expected to not only directly facilitate the spread of BVAB and other STIs, but also to do so indirectly via the positive feedback resulting from BV and STIs enhancing the spread of one another. BV for example has been shown to enhance susceptibility to chlamydia [17, 21], gonorrhoea [17], HIV [23, 90, 91], HSV-2 [18, 19] and *T. vaginalis* [17, 21, 22]. HSV-2 and *T. vaginalis* have in turn been found to increase the risk for development of BV and acquisition of other STIs [17, 20, 21, 23].

## Data from animal studies

Results from vaginal microbiome profiling in other animals are compatible with the hypothesis that differences in sexual network connectivity influence vaginal microbiomes. A study that compared the sympatric mice, *Peromyscus californius* and *Peromyscus maniculatus* found that the socially and genetically promiscuous *P. maniculatus* had greater vaginal microbiome diversity (a key feature of a BV-VMB) than the monogamous *P. californius* [92]. Likewise a study that compared the vaginal microbiomes of 9 primate species with different mating behaviors found that vaginal microbial diversity was strongly correlated with host-specific socioecologic factors such as female and male promiscuity [93]. Unlike the gut microbiome the vaginal microbiome showed little congruence with host phylogeny or diet [93]. The authors concluded that differences in sexual behavior were key determinants of the degree of vaginal microbial diversity.

### Empirical and simulation-based hypothesis tests

Testing the network-connectivity-VMB hypothesis would ideally involve longitudinal studies that follow up sympatric sub-populations with high and low STI prevalence from the time of sexual debut. Critically these studies should characterize the vaginal and penile microbiomes of sexual partners at frequent intervals. This, combined with detailed behavioral data, should enable researchers to ascertain if differences in network connectivity are responsible for the differential spread of BVAB in the high and low STI prevalence populations. The longitudinal study design should also provide better insights into the interactions between sexual behaviour, genital microbiomes and STIs. The importance of these longitudinal couple studies for the BV-network connectivity hypothesis cannot be overstated. We have shown evidence of a strong correlation between penile skin microbiota and the partner’s VMB. Longitudinal studies are however required to establish that these penile microbiota can be sexually transmitted to the man’s next partner and result in changes in her VMB.

In addition to empirical research, simulation-based study designs would also be useful to test aspects of the hypothesis presented here. Previous modeling studies have found that relatively small increases in network connectivity can lead to non-linear increases in HIV/STI spread [53]. If this applies to BV as well, then more connected sexual networks would be expected to facilitate the rapid spread of BVAB and various other STIs soon after sexual debut. Our own individual-based model of BVAB transmission provides a fitting illustration of the strong, non-linear effect of higher network connectivity, even in the absence of other STIs. The addition of a few key features could turn this didactic tool into a rigorous framework that unifies relevant knowledge of the microbiology, epidemiology and sociology of BV and other STI co-infections. In line with current insights from molecular microbiology, vaginal and penile microbiomes should be classified into at least five CSTs [43]. Furthermore, interactions with various STI co-infections – most notably chlamydia, gonorrhoea, *T. vaginalis*, HSV-2 and HIV – should be modelled explicitly, and other non-infectious causal factors (such as douching, smoking, diet) on the pathway to develop BV should be included as well. Lastly, the model should allow for more heterogeneity in sexual activity levels, as well as more structure in the network (for example, a non-random age-mixing pattern, and clustering of a high-risk core group within the network, and same sex partnerships). Besides the obvious advantages of being relatively fast and inexpensive, simulation studies can quantify the effect of uncertainty surrounding behavioral and biological parameters on the main outcomes measures.

If confirmed by empirical and simulated data, the network connectivity approach would offer a new paradigm for conceptualizing how differences in VMB emerge. If the proportion of a population that has BV is a population-level-property that is partially determined by network connectivity, then this introduces new options for prevention of BV and BV-associated adverse health outcomes such as adverse pregnancy outcomes and transmission of other STIs including HIV. It suggests that interventions that have been shown to reduce network connectivity may result in reductions in the prevalence of these VMBs. Because BV-VMBs may be responsible for a large proportion of the population attributable fraction of HIV and other STIs [16] and the spread of these STIs is also directly enhanced by network connectivity, small reductions in connectivity could translate into large declines in STI incidence. Uganda’s ‘Zero Grazing’ campaign [94] and similar processes elsewhere in Africa [95], which resulted in dramatic declines in side-partners and hence network connectivity, could be viewed as providing guidance for this approach. A better appreciation for the network connectivity would also help us unravel the disparities between ethnic groups that we see in BV, STIs and reproductive outcomes.

## Additional files

Additional file 1: A text document detailing the construction of the Netlogo model of BV transmission. (PDF 63 kb)

Additional file 3: The Netlogo model of BV transmission. (NLOGO 30 kb)

## Abbreviations

BV: Bacterial Vaginosis
CST: Community State Type
VMB: Vaginal Microbiome
