# Supplementary material for: Differential sexual network connectivity offers a parsimonious explanation for population-level variations in the prevalence of bacterial vaginosis: a data-driven, model-supported hypothesis
Source: BMC Womens Health. 2019 Jan 10;19:8. doi: 10.1186/s12905-018-0703-0 (PMC6327541; doi:10.1186/s12905-018-0703-0)
Supplement: Supplementary file 2 — A text document detailing the construction of the Netlogo model of BV transmission. (PDF 63 kb) [file 12905_2018_703_MOESM1_ESM.pdf]

Model description

This model description follows the structure as proposed by Grimm et al. (1)

Purpose

The purpose of this didactic model is to illustrate how differences in sexual network connectivity may explain variation in vaginal microbiome distributions, and the prevalence of Bacterial Vaginosis (BV). We want to simulate relationship formation and dissolution explicitly, as well as transitioning between the BV vaginal microbiome (BV-VMB) and other Community State Types (CSTs) of vaginal microbiota.

We divide the model world into two, entirely separate networks, one with higher and one with lower sexual connectivity.

Entities, state variables and scales

There are three types of entities in the model. The first are the men and women in the population. There are 2 types of individuals in the model ("breeds" in NetLogo lingo): LC (low connectivity) and HC (high connectivity) people.
The state variables of individuals are: age, the gender (male or female), the Community State Type (dichotomized into BV-VMB and other CSTs), the time since colonization with BV-VMB, the number of relationships an individual is engaged in, the person-specific probability of probability per time step to search for a partner (for men) and the person-specific probability of accepting a man's request to engage in a relationship (for women).

Strictly speaking, the square patches of land that the men and women are placed on are also entities of the model world, but geographical location does not play any role in this model. The model is run for up to 520 time steps, where each time step represents one week (i.e. we run the model for up to 10 year).

The third entity are the links that represent heterosexual relationships between individuals. Attributes of these links are the unique ID numbers of the two individuals forming the link, the time since the link was formed, and the expected time until the couple will break up.
In the graphical user interface, men and women are represented by stick figures in shades of blue and red respectively. A darker shade signifies that the individual is colonized with BV-VMB.

Process overview and scheduling

Each time step and for each breed:

- A fraction of the men form new relationships with women
- A fraction of couples break up
- Women and men may transition between BV-VMB and non-BV-VMB
- All individuals age one time step

Design concepts

- Basic principles: The basic principles addressed in this model are those of sexual network connectivity and the prevalence of BV-VMB.
- Emergence: The BV-VMB “epidemic”, measured by the prevalence of BV-VMB over time, emerges from the dynamics of CST transitions in the network.
- There is no adaptation, nor are there objectives or is there learning.
- Individuals “sense” (know) the relationship status and the age of their potential partners and the probability of relationship formation depends on these two properties.
- Couple formation and CST transitions are the only interactions between individuals. The probability of transitioning to BV-VMB depend on the CST of the partner(s).
- Stochasticity: stochasticity is used to represent uncertainty about who are the people who are colonized with BV-VMB at the start of the simulation (often called seed infections). Stochasticity is also used to represent variation in the processes of relationship formation, relationship break-up, and transition to BV-VMB.
- Observation: we are observing the distribution of BV-VMB as well as the point prevalence of partner concurrency and the ratio of the cumulative number of relationships formed in the high versus low connectivity networks at each time step.

Initialization

A closed population of men and women is initialized for each breed. At the start of the simulation, the age distribution is uniform between 15 and 40 years old. As the model is run for 10 years, the age distribution at the end of the simulation will be uniform between 25 and 50 years old. A random ten percent of the network is colonized with BV-VMB at the start of the simulation. for ease of visual representation and to limit computation time, we initiated the model with networks of only 250 people in the video clip and accompanying NetLogo file.

Input data

There are no external input data.

Submodels

Relationship formation

Men have a person-specific probability per time step to search for a partner. These probabilities are Beta distributed with shape parameters alpha = 3 and beta = 17, such that the average probability is 15% and the standard deviation around that is 7.8%. Searching is defined as identifying one potential partner, randomly, from all eligible partners who are within the allowable age range. This age range is set to up to 5 years above and below the man's age. In the low connectivity community, only single men search for partners, and eligible partners include only women who are also single. These restrictions do not apply in the high connectivity community. If the man finds a potential partner, she has a (person-specific) probability of accepting his request. These acceptance probabilities are again Beta

distributed with shape parameters alpha = 3 and beta = 17, such that the average probability is 15% and the standard deviation around that is 7.8%. If she accepts, the relationship is formed.

Relationship break-up
The duration of relationships is assumed to be log-normally distributed. When a new relationship is formed, the time until the relationship breaks up is drawn randomly from a log-normal distribution with a mean of 15 weeks and a standard deviation of 5 weeks.

CST transitioning
Men and women can transition from non-BV-VMB to BV-VMB with a given probability. This probability depends on the CST of the partner(s). In the absence of BV-VMB colonization in the partners (or if the individual has no partners during that time step), the probability of spontaneous transitioning from non-BV-VMB to BV-VMB is 0.1%. Each partner who is colonized with BV-VMB confers a risk of BV-VMB "transmission". In the default model, that risk is 20%. After a fixed period of time since the most recent transmission event (referred to as the colonization period), individuals with BV-VMB revert back to non-BV-VMB. In the reference model, this colonization period is much shorter in men (6 weeks) than in women (60 weeks). Note that partners can "reinfect" one another: In the special case of a 100% transmission risk, this means that the colonization period only starts after the relationship with the transmitting partner has ended. In general, it means that the colonization period gets reset each time an individual gets reinfected by (one of) his partner(s).

References

Grimm V, Berger U, DeAngelis DL, Polhill JG, Giske J, Railsback SF. The ODD protocol: A review and first update. Ecological Modelling; 221 (2010): 2760–2768. doi:10.1016/j.ecolmodel.2010.08.019
